# New diagnostic index for sarcopenia in patients with cardiovascular diseases

**DOI:** 10.1371/journal.pone.0178123

**Published:** 2017-05-18

**Authors:** Haruhito Harada, Hisashi Kai, Rei Shibata, Hiroshi Niiyama, Yasuhiro Nishiyama, Toyoaki Murohara, Noriko Yoshida, Atsushi Katoh, Hisao Ikeda

**Affiliations:** 1 Department of Cardiology, Kurume University Medical Center, Kurume, Japan; 2 Department of Advanced Cardiovascular Therapeutics, Nagoya University, Nagoya, Japan; 3 Department of Cardiology, Nagoya University, Nagoya, Japan; 4 Institute of Health and Sports Sciences, Kurume University, Kurume, Japan; 5 Department of Physical Therapy, Faculty of Fukuoka Medical Technology, Teikyo University, Omuta, Japan; Ehime University Graduate School of Medicine, JAPAN

## Abstract

**Background:**

Sarcopenia is an aging and disease-related syndrome characterized by progressive and generalized loss of skeletal muscle mass and strength, with the risk of frailty and poor quality of life. Sarcopenia is diagnosed by a decrease in skeletal muscle index (SMI) and reduction of either handgrip strength or gait speed. However, measurement of SMI is difficult for general physicians because it requires special equipment for bioelectrical impedance assay or dual-energy X-ray absorptiometry. The purpose of this study was, therefore, to explore a novel, simple diagnostic method of sarcopenia evaluation in patients with cardiovascular diseases (CVD).

**Methods:**

We retrospectively investigated 132 inpatients with CVD (age: 72±12 years, age range: 27–93 years, males: 61%) Binomial logistic regression and correlation analyses were used to assess the associations of sarcopenia with simple physical data and biomarkers, including muscle-related inflammation makers and nutritional markers.

**Results:**

Sarcopenia was present in 29.5% of the study population. Serum concentrations of adiponectin and sialic acid were significantly higher in sarcopenic than non-sarcopenic CVD patients. Stepwise multivariate binomial logistic regression analysis revealed that adiponectin, sialic acid, sex, age, and body mass index were independent factors for sarcopenia detection. Sarcopenia index, obtained from the diagnostic regression formula for sarcopenia detection including the five independent factors, indicated a high accuracy in ROC curve analysis (sensitivity 94.9%, specificity 69.9%) and the cutoff value for sarcopenia detection was -1.6134. Sarcopenia index had a significant correlation with the conventional diagnostic parameters of sarcopenia.

**Conclusions:**

Our new sarcopenia index using simple parameters would be useful for diagnosing sarcopenia in CVD patients.

## Introduction

Sarcopenia is an age-related syndrome characterized by progressive and generalized loss of skeletal muscle mass, weakening strength and decreasing physical performance with a risk of frailty that increases poor health outcomes, including falls, incident disability, hospitalization and mortality [1]. Sarcopenia can be categorized as ‘primary’(age-related) or ‘secondary’ (disease-related) sarcopenia. Disease-related sarcopenia is associated with advanced organ failure and chronic inflammatory diseases, such as chronic heart failure and chronic kidney disease secondary to cardiovascular diseases (CVD) [2]. In elderly patients with CVD, sarcopenia can be considered not only age-related, but also disease-related sarcopenia associated with CVD. Our previous article showed that sarcopenia with CVD was frequently found in elderly, female, small body mass index and chronic kidney disease patients [3].

Nutrition and chronic inflammation play an important role in the manifestation and progression of sarcopenia. The International Working Group on Sarcopenia recommended inflammation-related markers, oxidative stress markers, nutritional markers, antioxidant markers and hormones as biomarkers of sarcopenia [2]. For example, daily protein intake correlates with skeletal muscle index (SMI), handgrip strength and gait speed [3]. Adiponectin is an anti-inflammatory adipokine that is known to be associated with CVD and skeletal muscle function [4–8]. The conference report of ‘Cachexia in heart disease: highlights from the ESC 2010’ suggested that adiponectin might be a marker of muscle wasting in chronic heart failure [9].

The conventional diagnostic criteria of sarcopenia include loss of skeletal muscle mass, as assessed by the SMI, together with either weakened muscle strength, assessed by handgrip strength, or low physical performance, assessed by gait speed [2,10]. Although SMI is the most important component in the diagnosis of sarcopenia, it is difficult for general physicians to measure SMI routinely because the measurement requires either bioelectrical impedance assay or dual-energy X-ray absorptiometry. The purpose of this study was to explore a novel diagnostic method of sarcopenia assessment in patients with CVD, using simple parameters that do not need special equipment.

## Methods

### Study patients

We retrospectively studied 132 consecutive inpatients (age: 72±12 years, age range: 27–93 years, 80 males) with CVD and/or undergoing cardiovascular surgery who were admitted to our hospital between April 2013 and December 2015. These patients were divided into sarcopenic and non-sarcopenic groups. Patients with pacemaker implantation were excluded because bioelectric impedance assay could not be performed in them. Written informed consent was obtained from all patients, and the study was approved by the Ethics Committee of Kurume University.

### Diagnosis of sarcopenia

Sarcopenia was diagnosed by measuring muscle mass, muscle strength and physical performance according to the recommended diagnostic algorithm of the Asian Working Group for Sarcopenia (AWGS) guidelines [10]. Sarcopenia was defined as low SMI (< 7.0 kg/m^2^ in males; < 5.7 kg/m^2^ in females) associated with either low handgrip strength (< 26 kgf in males; < 18 kgf in females) or low gait speed (< 0.8m/sec). Non-sarcopenia was diagnosed when subjects had normal SMI (≥ 7.0 kg/m^2^ in males; ≥ 5.7 kg/m^2^ in females) or when they had normal handgrip strength (≥ 26 kgf in males; ≥ 18 kgf in females) and normal gait speed (≥ 0.8m/sec). The age criterion of more than 65 years old was not adopted for sarcopenia diagnosis in this study, because age is a possible confounding factor of disease-related (secondary) sarcopenia and, hence, CVD patients less than 65 years old were included in this study.

### Muscle mass measurements

Muscle mass was measured by bioelectrical impedance assay using the InBody S10 body composition analyzer (Biospace, Tokyo, Japan). This system applies electricity at frequencies of 1, 5, 50, 250, 500 kHz, and 1 MHz through the body. Whole-body impedance was measured using an ipsilateral foot-hand electrical pathway. The recommended conditions for bioelectrical impedance assay measurements, as explained to the subjects, were: (i) fasting for 4 hours before measurements; (ii) bladder voided before measurements; and (iii) no exercise in the 8-hour period prior to measurements [11]. Obviously edematous patients were examined after an improvement in their edema. Appendicular muscle mass was calculated as the sum of the muscle mass of the arms and legs. Absolute appendicular muscle mass was converted to SMI by dividing the value by the square of the height in meters (kg/m^2^).

### Muscle strength measurements

Muscle strength was assessed as handgrip strength using a Smedley hand dynamometer MY-2080 (Matsumiya Ikaseiki Seisakusho Co. Ltd., Tokyo, Japan). One trial was performed for each hand, and the result from the stronger hand was used for sarcopenia diagnosis.

### Physical performance measurements

Physical performance was assessed as usual gait speed. We referenced and modified a previous technique reported by Tanimoto et al [12]. Patients were asked to walk straight ahead for 12 meters at their usual speed for measurement of 10-meter walk time. The walking speed reached a steady speed within the first 2 meters. Gait speed (m/sec) was calculated by dividing the distance covered (10 meters (m)) by the 10-meter walk time (sec).

### Data collection

Data on admission were collected from hospital charts and databases. Blood was drawn from the antecubital vein early in the morning, after at least 8-hours of fasting, and subjected to biochemical and immunological measurements, including high sensitivity C-reactive protein (hsCRP), interleukin-6 (IL-6), sialic acid and adiponectin. General nutritional condition was assessed using body mass index (BMI) and the controlling nutritional status (CONUT) score, which was determined using serum albumin, total cholesterol and total lymphocyte count [13].

### Statistical analysis

All data were statistically analyzed using the Ekuseru-Toukei 2012 software purchased from Social Survey Research Information Co., Ltd. (Tokyo, Japan). Numerical data were expressed as mean ± standard deviation or as median with 95% confidence interval (CI). Student’s t-test and Mann-Whitney u-test were appropriately used to compare the two groups. Proportional data was analyzed using chi-square test or Fisher’s exact test. Univariate and stepwise multivariate logistic regression analyses were performed to determine independent factors for sarcopenia diagnosis. Receiver operating characteristic (ROC) curve analysis was calculated to predict the cutoff value for sarcopenia diagnosis. Statistical significance was considered at the level of p<0.05.

## Results

### Characteristics of sarcopenic patients

As shown in Table 1, 39 of 132 patients (29.5%) were diagnosed as having sarcopenia. Sarcopenic patients were older and had lower estimated glomerular filtration rates (eGFR) and lower Barthel index, and included higher numbers of females and subjects with chronic kidney disease as compared with non-sarcopenic patients. The distributions of eGFR and Barthel index were not biphasic in sarcopenia and non-sarcopenia groups (S1 Fig). The prevalence of symptomatic heart failure, baseline CVD, comorbidities and left ventricular ejection fraction did not differ between the two groups. SMI, handgrip strength and gait speed were significantly lower in sarcopenic patients than those in non-sarcopenic patients (Table 2).

**Table 1:** Baseline characteristics of sarcopenic and non-sarcopenic patients.

	Non-sarcopenic n = 93	Sarcopenic n = 39	p value
Age, years, (range)	70 ± 13 (27–93)	77 ± 8 (54–92)	0.0001
Male, n (%)	67 (72.0)	13 (33.3)	< 0.0001
Symptomatic heart failure (ACC/AHA CHF stage C and D), n (%)	27 (29.0)	16 (41.0)	0.2226
Cardiovascular disease, n (%)			
CAD (including CABG)	53 (57.0)	17 (43.6)	0.1837
Hypertensive heart disease	8 (8.6)	5 (12.8)	0.5252
Idiopathic cardiomyopathy	6 (6.5)	2 (5.2)	1.0000
Arrhythmia	12 (12.9)	3 (7.7)	0.5513
Valvular heart disease	9 (9.7)	8 (20.5)	0.1514
Peripheral artery disease	3 (3.2)	2 (5.1)	0.6319
PCS (excluding CABG)	2 (2.2)	2 (5.1)	0.5812
Comorbidity, n (%)			
Hypertension	60 (64.5)	24 (61.5)	0.8432
Diabetes mellitus	37 (39.8)	12 (30.8)	0.4300
Dyslipidemia	34 (36.6)	13 (33.3)	0.8427
Hyperuricemia	9 (9.7)	3 (7.7)	1.0000
Chronic kidney disease	10 (10.8)	11 (28.2)	0.0184
Stroke	11 (11.8)	2 (5.1)	0.3432
eGFR, mL/min/1.73m^2^	64.3 (62.1–71.0)	51.2 (41.0–59.0)	0.0028
Left ventricular ejection fraction, %	64.0 (62.0–67.0)	62.0 (59.0–65.0)	0.1494
Barthel index	100 (90–100)	90 (85–100)	0.0171

Numerical data are expressed as mean ± standard deviation or as median (95% confidence interval). The numbers in parenthesis denote the percentage. ACC/AHA CHF stage = American College of Cardiology/American Heart Association chronic heart failure stage; CABG = coronary artery bypass grafting; CAD = coronary artery disease; eGFR = estimated glomerular filtration rate; PCS = post-cardiovascular surgery.

**Table 2:** Baseline values of the diagnostic components for sarcopenia in sarcopenic and non-sarcopenic patients.

	Non-sarcopenic	Sarcopenic	p value
Skeletal muscle index, kg/m^2^	7.36 ± 1.24 (n = 85)	5.20 ± 0.71 (n = 39)	<0.0001
Handgrip strength, kgf	30.0 ± 12.7 (n = 69)	12.6 ± 5.9 (n = 34)	<0.0001
Gait speed, m/sec	1.16 ± 0.40 (n = 58)	0.75 ± 0.27 (n = 26)	<0.0001

Numerical data are expressed as mean ± standard deviation.

### Muscle-related inflammatory and nutritional markers

Serum levels of IL-6, adiponectin and sialic acid were significantly higher in sarcopenic than in non-sarcopenic patients (p<0.01, p<0.0001 and p<0.05, respectively) (Table 3). There was no significant difference in hsCRP between the 2 groups. Sarcopenic patients had significantly lower BMI and serum albumin than non-sarcopenic patients (p<0.0001 and p<0.05, respectively). In total cholesterol, total lymphocyte count and CONUT score, there were no significant differences between sarcopenic and non-sarcopenic groups.

**Table 3:** Muscle-related inflammatory and nutritional markers.

	Non-sarcopenic n = 93	Sarcopenic n = 39	P value
Muscle-related inflammatory markers			
hs-CRP, ng/mL	1280 (907–2290)	2440 (915–6970)	0.1913
Interleukin-6, pg/mL	4.20 (3.50–4.80)	5.70 (4.30–10.10)	0.0072
Adiponectin, μg/mL	3.61 (3.22–4.50)	8.32 (6.52–11.50)	<0.0001
Sialic acid, mg/dL	62.0 (59.0–65.0)	65.0 (60.0–76.0)	0.0247
Nutritional markers			
Body mass index, kg/m^2^	25.6 ± 4.8	21.7 ± 2.5	<0.0001
Serum albumin, g/dL	4.11 ± 0.46	3.93 ± 0.39	0.0332
Total cholesterol, mg/dL	173 ± 45	170 ± 31	0.7061
Total lymphocyte count, /μL	1540 ± 689	1333 ± 542	0.0972
CONUT	2.0 (1.0–2.0)	2.0 (2.0–3.0)	0.2132

Data were expressed as mean ± standard deviation or as median (95% confidence interval). CONUT = controlling nutritional status score; hs-CRP = high sensitivity c-reactive protein.

### Sarcopenia index

To determine the index for diagnosing sarcopenia, binomial logistic regression analyses for the presence of sarcopenia were performed in the entire patient cohort, obtained by assessing sarcopenic and non-sarcopenic groups together. Univariate binomial logistic regression analyses revealed that age (positively), female gender (positively), adiponectin (positively), sialic acid (positively), BMI (inversely), albumin (inversely), and eGFR (inversely) significantly correlated with sarcopenia (Table 4). In stepwise multivariate binomial logistic regression analysis using the above 7 significant variables, adiponectin, sialic acid, age, BMI and sex were independent factors for sarcopenia (Table 5). According to the partial regression coefficients of the independent factors, the following diagnostic regression formula is considered as a diagnostic index for sarcopenia:

**Table 4:** Single binomial logistic regression analysis.

Biomarkers	SE	β	p value
Age	0.0223	0.8214	0.0023
Sex, (male = 1, female = 2)	0.4108	0.8012	0.0001
Estimated glomerular filtration rate	0.0093	—0.4546	0.0294
Muscle-related inflammatory markers			
Adiponectin	0.0410	0.7844	0.0001
Sialic acid	0.0180	0.5424	0.0057
High-sensitivity C reactive protein	0.1196	0.2998	0.1224
Interleukin-6	0.0175	0.1217	0.4973
Nutritional markers			
Body mass index	0.0761	—1.5047	0.0001
Albumin	0.4494	—0.4081	0.0398
Controlling nutritional status	0.1063	0.1127	0.5481
Total lymphocyte count	0.0003	—0.3326	0.0990
Total cholesterol	0.0046	—0.0627	0.7435

β = standardized partial regression coefficient; SE = standard error.

**Table 5:** Stepwise multivariate binomial logistic regression analysis for sarcopenia.

Independent variables	Partial regression coefficient	SE	β	p value
Body mass index	—0.3430	0.1034	—1.5674	0.0009
Sialic acid	0.0840	0.0287	0.9121	0.0034
Sex, (male = 1, female = 2)	1.5751	0.5457	0.7696	0.0039
Age	0.0843	0.0328	1.0212	0.0101
Adiponectin	0.1117	0.0563	0.5551	0.0472
Constant term	—7.8072	3.9208	-	0.0465

β = standardized partial regression coefficient; SE = standard error. There were no significant correlations for the following variables: estimated glomerular filtration rate and albumin.

Sarcopenia index = 0.1117 x [adiponectin (μg/mL)] + 0.0840 x [sialic acid (mg/dL)] + 1.5751 x [sex (male = 1, female = 2)] + 0.0843 x [age (years)]—0.3430 x [BMI (kg/m^2^)]—7.8072.

### Sarcopenia index and conventional diagnostic criteria for sarcopenia

**Fig 1:**
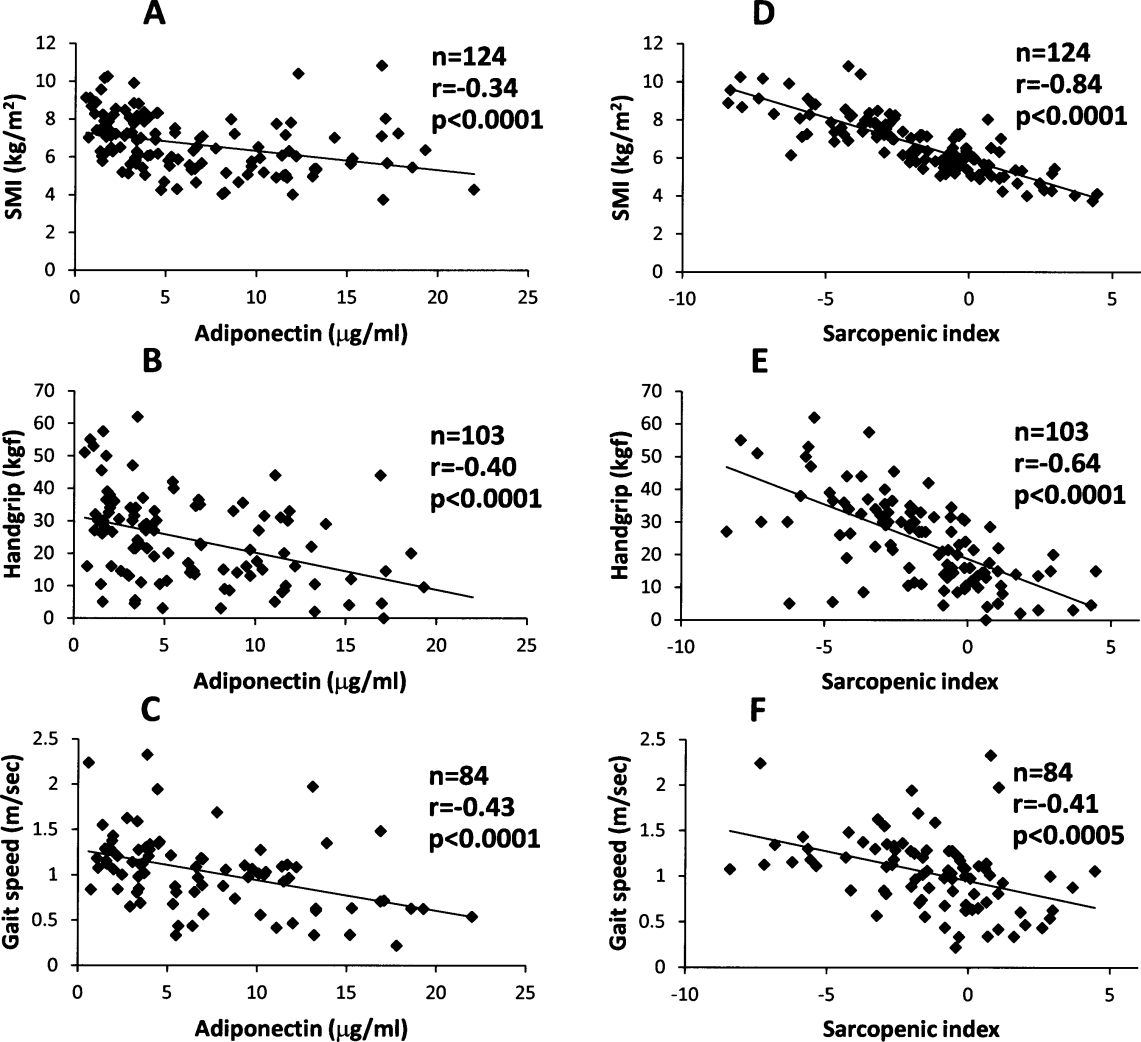
**Correlations of adiponectin (A, B and C) and sarcopenia index (D, E and F) with skeletal muscle index (A and D, n = 124), handgrip strength (B and E, n = 103), and gait speed (C and F, n = 84) in patients with cardiovascular disease.** r = correlation coefficient. Sarcopenia index = 0.1117 x [adiponectin (μg/mL)] + 0.0840 x [sialic acid (mg/dL)] + 1.5751 x [sex (male = 1, female = 2)] + 0.0843 x [age (years)]—0.3430 x [BMI (kg/m^2^)]—7.8072.

As shown in Fig 1A, 1B and 1C, adiponectin had weak negative correlations with SMI (r = -0.34) and handgrip strength (r = -0.40), and a modest correlation with gait speed (r = -0.43), whereas sialic acid weakly correlated with only SMI (r = -0.21) (S2 Fig). In contrast, the sarcopenia index showed a strong negative correlation with SMI (r = -0.84), and modest negative correlations with handgrip strength (r = -0.64) and gait speed (r = -0.41) (Fig 1D, 1E and 1F).

### Cutoff value of sarcopenia index for sarcopenia diagnosis

**Fig 2:**
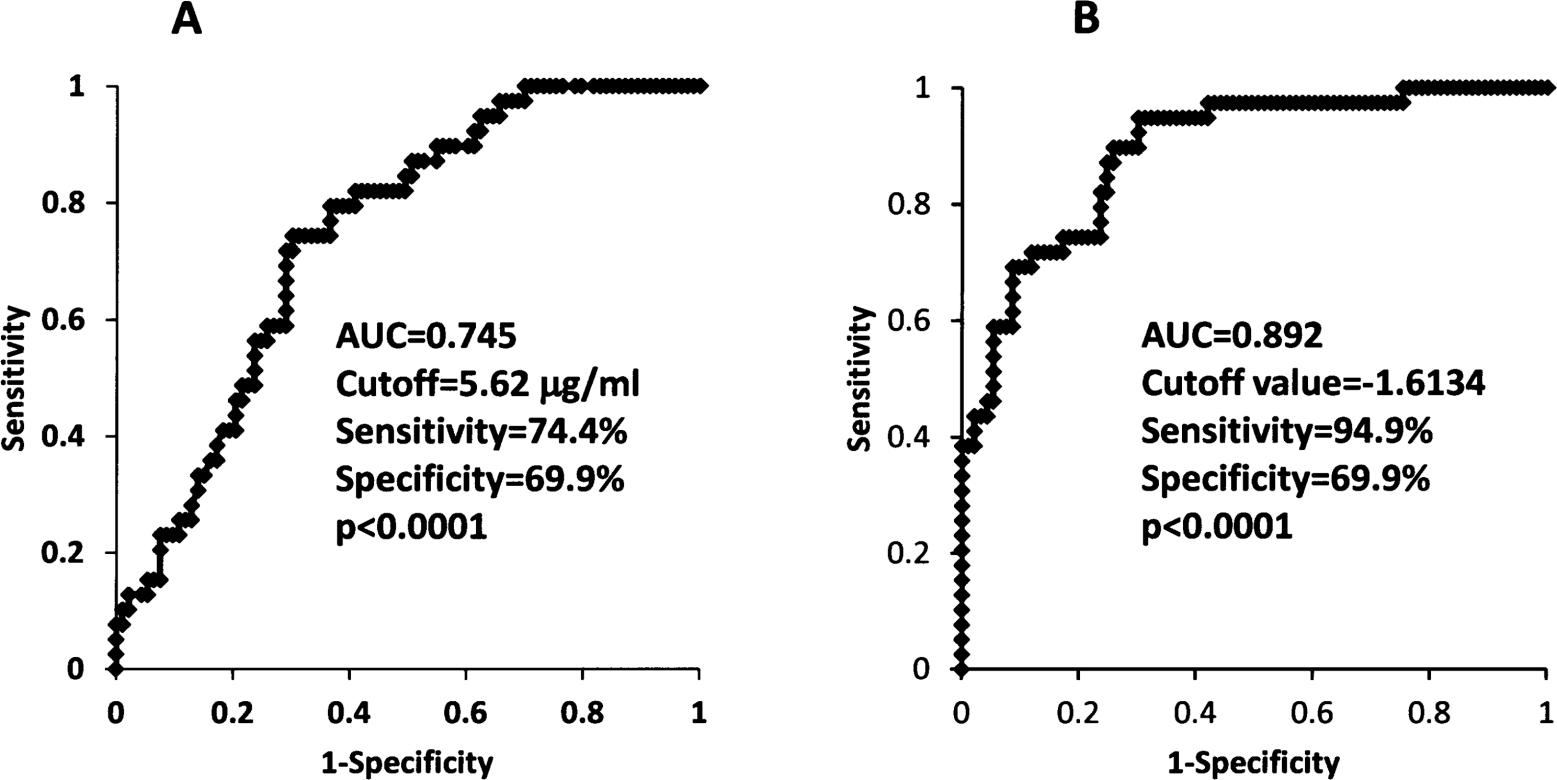
**Receiver operating characteristic curve analyses of adiponectin (A) and sarcopenia index (B) for the detection of sarcopenia in patients with cardiovascular diseases.** AUC = area under the curve.

In ROC curve analysis, the cutoff value of adiponectin for detection of sarcopenia was 5.62 μg/ml, with a sensitivity and specificity of 74.4% and 69.9%, respectively (Fig 2A). In contrast, sarcopenia index significantly improved the diagnostic accuracy for sarcopenia: ROC analysis showed that the diagnostic accuracy of the sarcopenia index had a sensitivity of 94.9% and specificity of 69.9%. The cutoff value for sarcopenia detection was -1.6134 (Fig 2B).

## Discussion

In this study, we proposed the sarcopenia index as a new diagnostic tool for sarcopenia in CVD patients, which is independent of the conventional diagnostic criteria, i.e. skeletal muscle mass, handgrip force and gait speed. The sarcopenia index is calculated by a regression formula including the five components of age, sex, BMI, serum adiponectin and serum sialic acid, which are independent factors for detecting sarcopenia, based on stepwise multivariate binomial logistic regression analysis.

BMI, which represents age and nutritional status, is an important indicator of the progression and manifestation of sarcopenia, as previously reported [2]. Generally, elderly people more than 65 years old are the subjects of studies on the diagnosis of primary sarcopenia [2,10]. We excluded the age criterion in this study because age was expected to be a possible confounding factor in disease-related sarcopenia secondary to CVD, and hence, CVD patients less than 65 years old were included in this study. Indeed, stepwise multivariate binomial logistic regression analysis revealed that age was one of the independent factors for sarcopenia diagnosis in CVD patients, as shown in Table 5. Since the diagnostic criteria of SMI are reportedly different between males and females [2,10], and the proportion of females with sarcopenia was higher than those in the non-sarcopenia group, as shown in Table 1, gender cannot be ignored when diagnosing sarcopenia.

Elevation of the concentration of adiponectin has been reported as a marker of weakened skeletal muscle force [6,7]. On the other hand, however, adiponectin is known to protect the skeletal muscle against inflammation and injury in dystrophinopathies [14]. Also, in in vitro analyses, treatment with globular adiponectin induced differentiation and fusion of skeletal muscle cells [15], and activated motility and regenerative traits in muscle satellite cells [16]. Hence, the up-regulation of adiponectin in sarcopenia may be a compensatory response to the stress of muscle wasting, as shown in functional overloading-associated muscle hypertrophy or regrowth of unloading-associated atrophied muscle [17].

Sialic acid, a derivative of neuraminic acid, has multiple physiological functions. Sialic acid is necessary for maintenance of skeletal muscle motor performance [18,19], and loss of sialic acid leads to myopathy [20]. The concentration of sialic acid in muscle is reportedly a marker of skeletal muscle aging [21]. We speculate that sialic acid may be up-regulated to compensate for reduced skeletal muscle motor performance in sarcopenia. Accordingly, it is considered that both adiponectin and sialic acid play an important role in skeletal muscle function and maintenance.

It was noteworthy that the sarcopenia index more strongly correlated with the three conventional diagnostic components of sarcopenia as compared with serum adiponectin, which is an established biomarker of sarcopenia [4–8], as shown in Fig 1. Moreover, ROC curve analysis demonstrated that the sarcopenia index had a greater AUC and higher sensitivity for detecting sarcopenia than serum adiponectin (Fig 2). Taken together, this suggests that sarcopenia index could be a new, accurate and reliable diagnostic tool for evaluation of sarcopenia in CVD patients.

Further, the sarcopenia index can be calculated by measurement of adiponectin and sialic acid and simple physical data (age, sex, weight and height). Since specific equipment and skill are required to evaluate the conventional diagnostic criteria, such as skeletal muscle mass and physical performance (handgrip strength and gait speed), it is sometimes difficult for general physicians to diagnose sarcopenia in their daily clinical practice. Therefore, sarcopenia index may have an advantage over conventional diagnostic methods in the screening of sarcopenia in CVD patients.

### Limitations

The first limitation of this study was the small number of subjects and biomarkers investigated. Future large-scaled studies investigating a greater spectrum of biomarkers will improve the sensitivity and specificity of sarcopenia index. Next, we focused on CVD-related sarcopenia in this study. It is possible that the sarcopenia index for CVD-related sarcopenia might not be applicable for ‘primary’ (or age-related) sarcopenia and ‘secondary‘ sarcopenia related to other diseases. Second, as shown in new S1 and S2 Tables, the characteristic features of the baseline characteristics, nutritional markers and circulating levels of inflammatory markers in the females were almost similar to those in the whole patient group, whereas the characteristic features in the males seemed different from those in the females. Thus, it is possible that the gender difference would be important issue. The gender difference of the sarcopenia index should be addressed in future study including larger number of patients with enough statistic power.

### Conclusion

We proposed a new diagnostic index for sarcopenia in CVD patients, which is calculated using values of circulating concentrations of adiponectin and sialic acid and simple physical data (i.e. sex, age, height and body weight). We believe that this sarcopenia index could be a simple and useful tool for diagnosing sarcopenia in CVD patients.

## Supporting information

S1 Fig Histogram of number of patient in Barthel index and estimated glomerular filtration rate. eGFR = estimated glomerular filtration rate.
(TIFF)

S2 Fig Correlations of sialic acid and skeletal muscle index. SMI = skeletal muscle index.
(TIFF)

S1 Table Baseline characteristics of sarcopenic and non-sarcopenic patients in each gender. (TIF)

S2 Table Muscle-related inflammatory and nutritional markers in each gender. (TIF)
