# Supplementary figures and images for: New diagnostic index for sarcopenia in patients with cardiovascular diseases
Source: PLoS One. 2017 May 18;12(5):e0178123. doi: 10.1371/journal.pone.0178123 (PMC5436875; doi:10.1371/journal.pone.0178123)

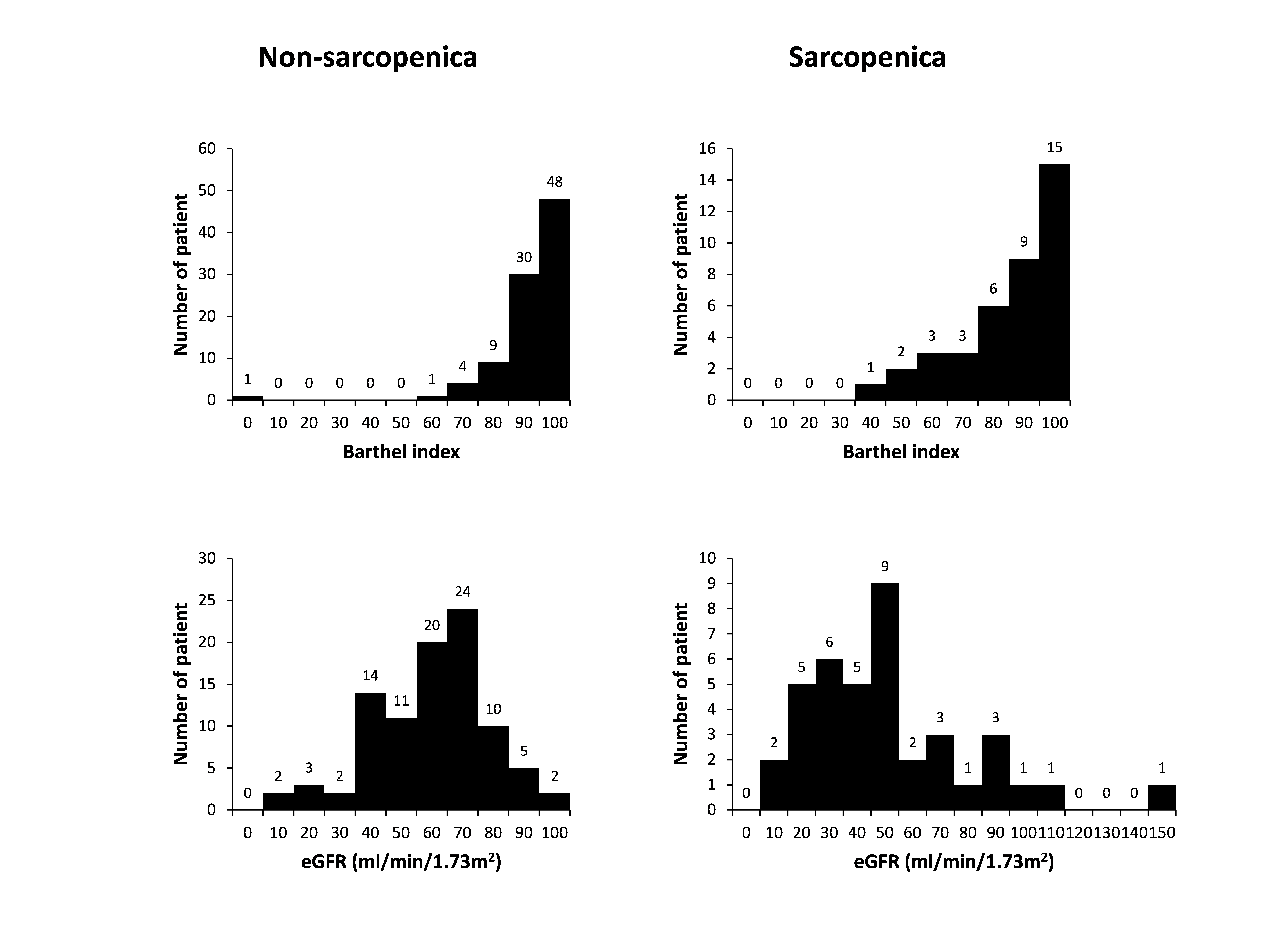

Supplement: S1 Fig — eGFR = estimated glomerular filtration rate. (TIFF) [file pone.0178123.s001.tiff]

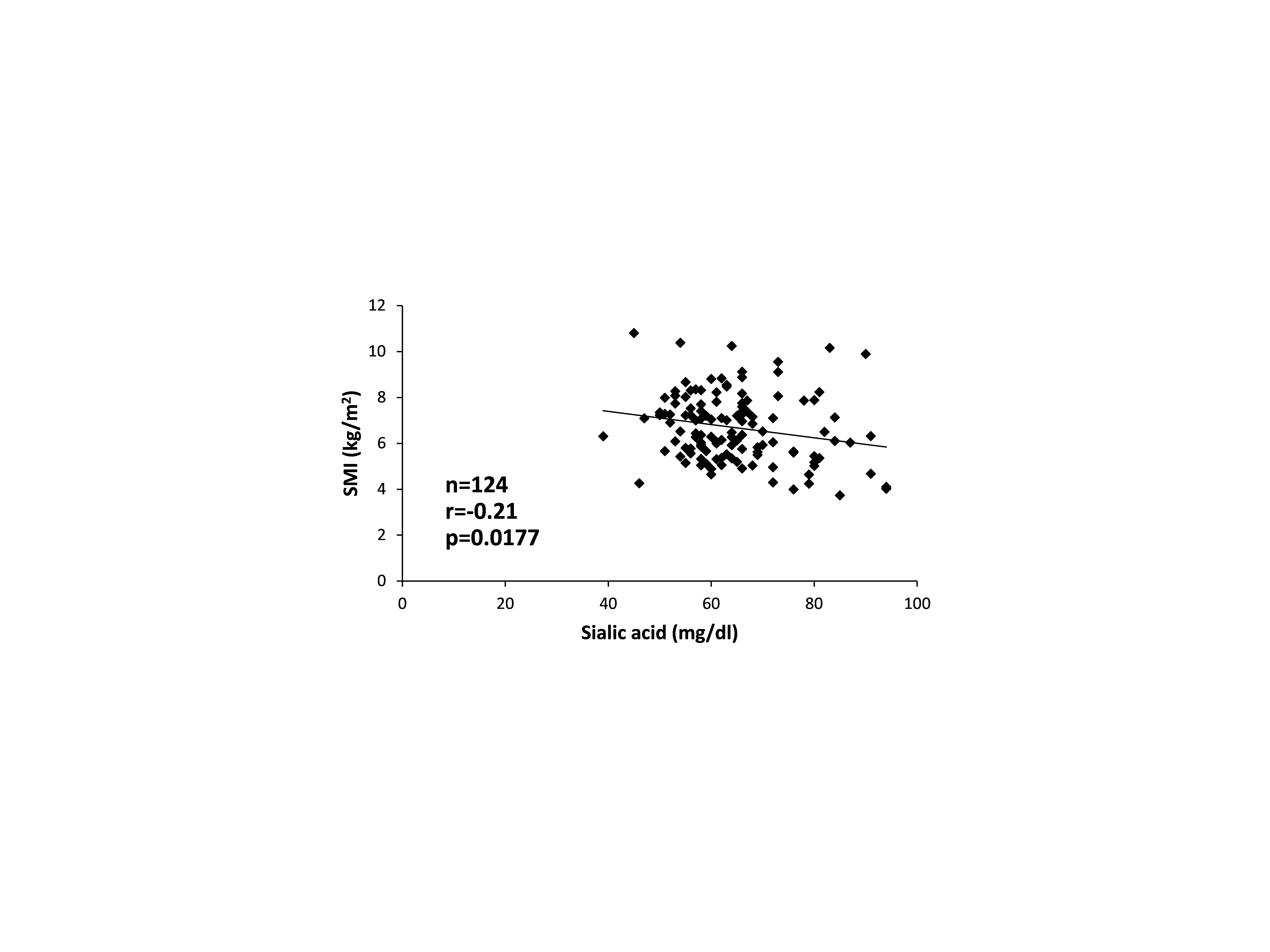

Supplement: S2 Fig — SMI = skeletal muscle index. (TIFF) [file pone.0178123.s002.tiff]

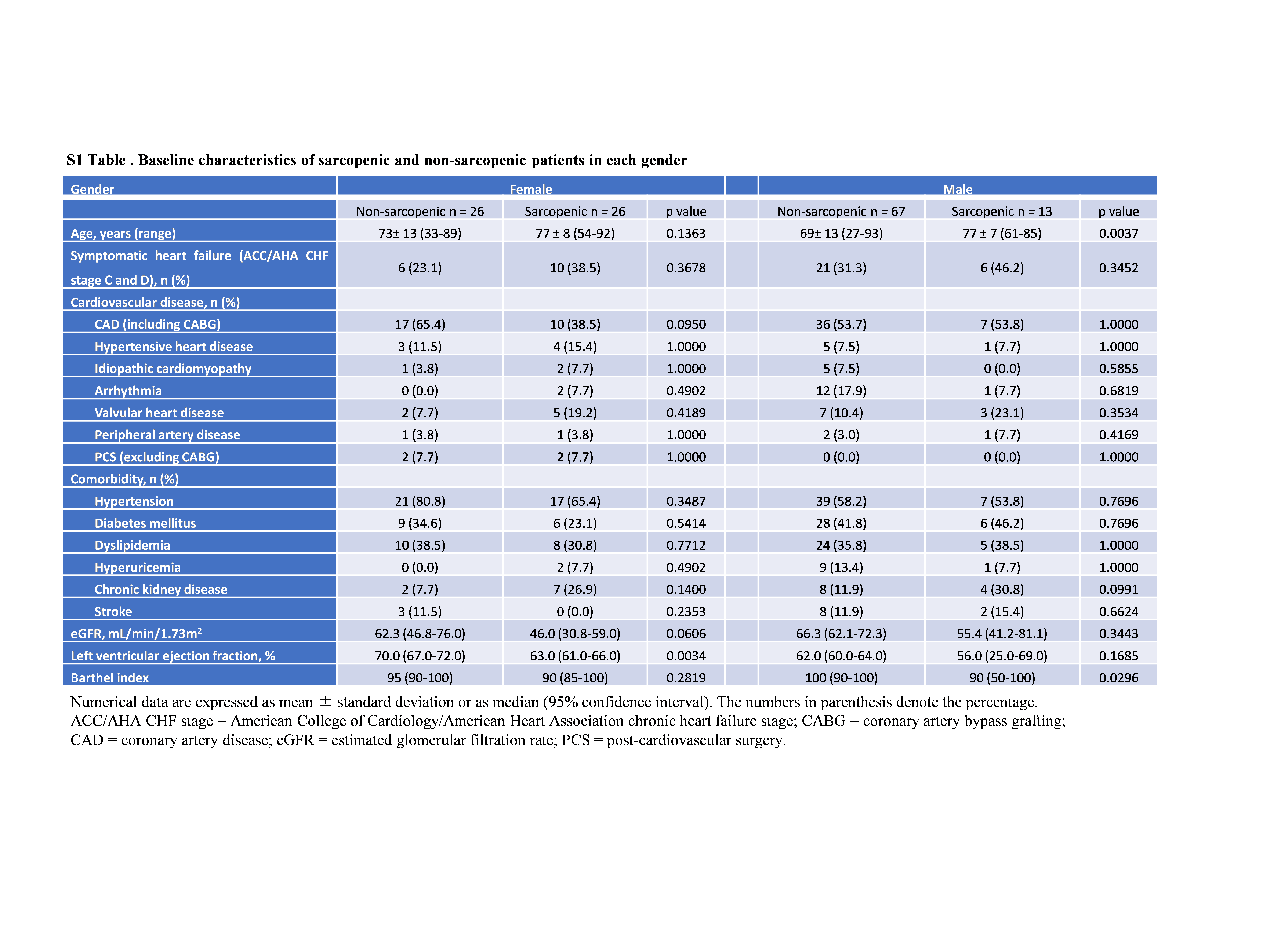

Supplement: S1 Table — (TIF) [file pone.0178123.s003.tif]

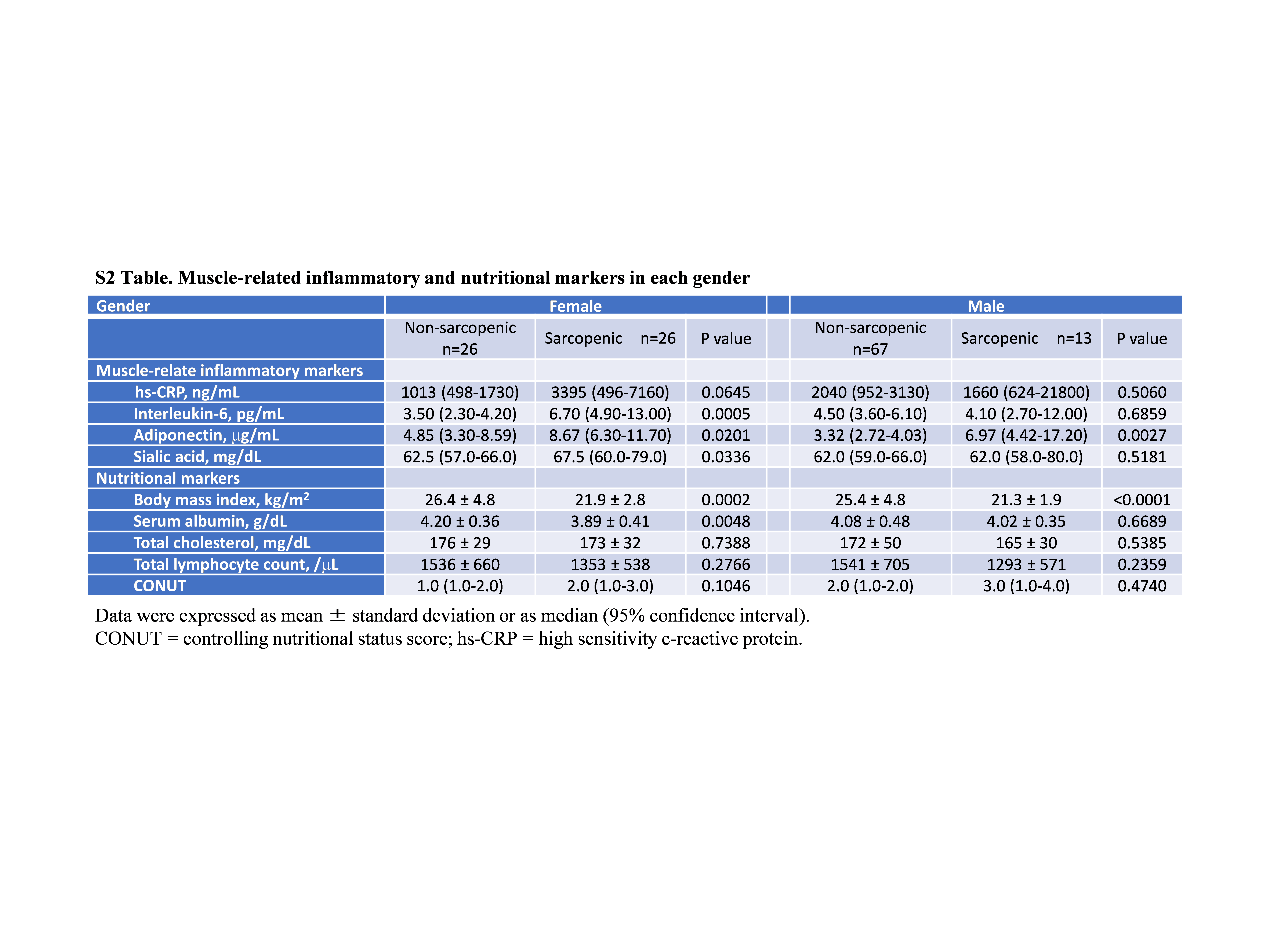

Supplement: S2 Table — (TIF) [file pone.0178123.s004.tif]
